# Natural Perylenequinone Compounds as Potent Inhibitors of *Schistosoma mansoni* Glutathione S-Transferase

**DOI:** 10.3390/life13101957

**Published:** 2023-09-25

**Authors:** Benson Otarigho, Mofolusho O. Falade

**Affiliations:** 1 Department of Molecular Microbiology and Immunology, Oregon Health and Science University, Portland, OR 97239, USA; 2 Department of Biology, Transylvania University, Lexington, KY 40508, USA

**Keywords:** schistosomiasis, antischistosomal agents, glutathione S-transferase (GST), parasitic worms, natural compounds, molecular docking, *Caenorhabditis elegans*, perylenequinones

## Abstract

The existing treatment strategy for Schistosomiasis centers on praziquantel, a single drug, but its effectiveness is limited due to resistance and lack of preventive benefits. Thus, there is an urgent need for novel antischistosomal agents. *Schistosoma* glutathione S-transferase (GST) is an essential parasite enzyme, with a high potential for targeted drug discovery. In this study, we conducted a screening of compounds possessing antihelminth properties, focusing on their interaction with the *Schistosoma mansoni* glutathione S-transferase (*Sm*GST) protein. We demonstrated the unique nature of *Sm*GST in comparison to human GST. Evolutionary analysis indicated its close relationship with other parasitic worms, setting it apart from free-living worms such as *C. elegans*. Through an assessment of binding pockets and subsequent protein–ligand docking, we identified Scutiaquinone A and Scutiaquinone B, both naturally derived Perylenequinones, as robust binders to *Sm*GST. These compounds have exhibited effectiveness against similar parasites and offer promising potential as antischistosomal agents.

## 1. Introduction

Schistosomiasis is a debilitating tropical disease caused by *Schistosoma* parasites, posing a significant burden on global health, particularly in regions with poor access to sanitation and clean water [1,2,3,4]. Schistosomiasis affects millions of people in developing countries, particularly in sub-Saharan Africa, Asia, and South America [1,3,4,5]. *Schistosoma mansoni* is one of the species responsible for causing schistosomiasis in humans. Despite efforts to control and eliminate the disease, schistosomiasis remains a significant global health concern [1,2,3,4,5]. Current treatment options primarily rely on a limited number of drugs, such as praziquantel, which has been effective in reducing morbidity and controlling the spread of the disease. However, the continuous reliance on praziquantel has led to concerns about the potential emergence of drug-resistant strains of *Schistosoma*, posing a serious threat to the efficacy of this drug and the overall management of the disease [6,7,8,9,10,11]. On the other hand, developing a vaccine against *Schistosoma* parasites has been a challenging endeavor due to the complex life cycle of these parasites and the way they interact with the human immune system. Therefore, there is currently no vaccine available to prevent schistosomiasis. Considering the scarcity of verified drug targets for schistosomes, compounds with distinct mechanisms of action from praziquantel represent a valuable asset in the battle against these parasites and offer new therapeutic strategies to combat schistosomiasis effectively [12,13,14]. One promising avenue of research involves targeting a key enzyme present in the parasite, *Schistosoma mansoni* Glutathione S-Transferase (*Sm*GST) [15]. *Sm*GST is crucial for the parasite’s survival as it helps the worm neutralize and eliminate harmful molecules, including reactive oxygen species and toxic xenobiotics, which can be detrimental to its cellular functions. By targeting this enzyme with specific inhibitors, it is possible to disrupt the detoxification process, leading to an accumulation of toxic molecules within the parasite, impairing its redox homeostasis, and ultimately causing its death [16,17].

In further response to the challenges associated with the utilization of praziquantel, scientists have redirected their focus towards other synthetic agents and, notably, natural compounds sourced from diverse origins like plants, fungi, and marine organisms [18,19,20,21]. These natural compounds have long been recognized for their diverse biological activities and have been extensively studied for their potential medicinal applications [19,22]. However, the development of natural compounds as antischistosomal agents is not without challenges [23]. One significant obstacle is the limited availability of some of these compounds, as they might be found in only specific plant species or organisms [19,22]. Additionally, the low bioavailability of certain natural compounds can hinder their effective delivery to the target sites in the parasite. Besides formulation strategies, structural optimization and molecular docking of these compounds against targeted proteins are necessary to enhance the pharmacological properties and bioavailability of the compounds [24,25]. Molecular docking of natural compounds offers a compelling and innovative approach in the treatment of diseases such as Schistosomiasis [26,27]. Hence, docking that results in strong affinity binding could target *Schistosoma mansoni*-specific proteins, thereby disrupting detoxification processes, impairing redox homeostasis, and weakening the parasite’s survival mechanisms [28]. Moreover, the multifaceted nature of these compounds, encompassing antioxidant, anti-inflammatory, and immunomodulatory properties, presents a comprehensive therapeutic strategy against schistosomiasis [29]. 

A compelling approach in drug development involves targeting enzymes specific to parasites, distinguishing them from their human homologs. In the current study, we also assessed the similarity between *Sm*GST and its human homologs. The evolutionary relationship of *Sm*GST with other parasitic worms and free-living worms like *Caenorhabditis elegans* was also explored. Protein–ligand docking studies demonstrated that Scutiaquinone A and Scutiaquinone B, natural compounds categorized as Perylenequinones, exhibited strong binding affinities to *Sm*GST. These compounds have the potential to serve as potent antischistosomal agents, targeting the parasite’s GST enzyme. This specificity offers promising advantages for targeted treatments against schistosomiasis, addressing current therapeutic challenges, and advancing the fight against this disease. 

## 2. Methods

### 2.1. Exploring and Extracting Antihelminth Compounds and SmGST Protein Sequences from Databases

One of the objectives of this study was to explore and identify compounds that exhibit anthelmintic activities. To achieve this goal, we conducted an extensive search across multiple databases renowned for their comprehensive collection of chemical and biological information. The databases used for this purpose included SANCDB [30,31], which specializes in natural compounds; PubChem [32,33], a vast repository of chemical structures and biological activities; and DrugBank [34], a comprehensive database of drugs and their associated properties. We obtained the relevant data from these databases, and the compounds of interest were extracted in the Protein Data Bank (PDB) format, which allowed for us to analyze and visualize the atomic arrangements of the compounds, which can provide valuable insights into their interactions with biological targets. To facilitate further analysis and make the findings accessible, we meticulously documented common names and accessions or unique identifiers of the results in a tabular form (Table 1). To conduct molecular docking studies involving the extracted compounds and *Sm*GST, the protein’s sequence and structure (*Sm*GST) were acquired from the Protein Database, accessible at https://www.rcsb.org/, (accessed on 10 June 2023) using the accession number 1U3I.

### 2.2. Alignment of Schistosoma mansoni GST Protein Sequences to Mammalian Homologs 

Given that *Schistosoma* parasites are known to infect a wide range of hosts, we aimed to investigate the similarity of the *Sm*GST protein sequences with GST sequences from mammals and other vertebrates. To achieve this, we used the BLASTP program, which is available on the NCBI BLAST (Basic Local Alignment Search Tool) platform [35]. We used the *Sm*GST protein sequences as the query and searched against a dataset of GST protein sequences from mammals and other vertebrates. The BLASTP analysis allowed for us to identify similar sequences in the mammalian and vertebrate datasets that shared significant homology with the *Sm*GST sequences. These similar sequences might indicate potential conserved functions or regions among different organisms. After obtaining similar protein sequences from the BLASTP search, the next step was to perform a multiple sequence alignment, which was subjected to a phylogenetic tree analysis using MEGA [36,37].

### 2.3. Schistosoma mansoni GST Relationship to Other Parasitic Worms as Well as Caenorhabditis elegans

We also aimed to analyze the similarity between *SmGST* and its homologs in other helminths. To accomplish this, we conducted a BLAST search of the *SmGST* protein against a range of worm parasites, including liver flukes (*Clonorchis sinensis*, *Fasciola hepatica,* and *Opisthorchis viverrine*), which cause liver diseases; lung flukes (*Paragonimus westermani*), which cause lung infections; intestinal flukes (*Fasciolopsis buski*), which cause intestinal disorders; tapeworms (*Taenia solium*, *Echinococcus granulosus*, and *Echinococcus multilocularis*), which cause cystic diseases; hookworms (*Necator americanus*, *Ancylostoma duodenale*, and *Ancylostoma ceylanicum*), which cause anemia and malnutrition; and a free-living worm (*Caenorhabditis elegans*), which is a model organism for studying biology. We obtained the GST protein sequences of these worms from the NCBI database, which is a public repository of biological data. We then used ClustalW version 2 (https://www.ebi.ac.uk/Tools/msa/clustalw2/) (accessed on 13 June 2023), which is a software tool that aligns multiple sequences based on their similarities and differences, to perform a multiple alignment of the GST sequences. This allowed us to identify regions of conservation and variation among the GST sequences, as well as possible functional or structural domains. We then used the MEGA software version 11.0.10, which is a software tool that infers evolutionary relationships among sequences based on various methods and models, to construct a phylogenetic tree of the GST sequences. This allowed for us to delineate possible common ancestries and divergences among the worms.

### 2.4. Protein–Ligand Docking of Compounds against Schistosoma mansoni GST

The compounds obtained from the previous step were subjected to molecular docking against the *Sm*GST proteins using the CB-Dock2 server, accessible at https://cadd.labshare.cn/cb-dock2/php/blinddock.php (accessed on 20 June 2023) [38]. CB-Dock2 is an advanced protein–ligand blind docking server that incorporates improvements in binding site identification and binding pose prediction. The CB-Dock2 server utilizes a combination of techniques to enhance the accuracy of the docking process. It integrates cavity detection, docking algorithms, and homologous template fitting to improve the reliability of the docking predictions. By employing these techniques, CB-Dock2 aims to provide more accurate predictions of protein–ligand interactions. The blind docking approach implemented by CB-Dock2 allows for the exploration of potential binding sites on the target proteins without prior knowledge of their exact locations. This enables the identification of potential binding pockets that may not be readily apparent based solely on the protein’s structure.

We submitted the PDB file of the SmGST protein and the MOL2 file of the compounds to CB-Dock2. During the docking process, the compounds were systematically evaluated for their binding affinity with the target proteins. The potential binding pockets were subsequently validated using the DrugRep tool (http://cao.labshare.cn/drugrep/) (accessed on 29 June 2023). This tool facilitates the virtual screening of drug libraries, encompassing FDA-approved, experimental, and traditional Chinese medicine compounds, against specific selected binding pockets of the target protein. Its capabilities include an automatic molecular 3D structure construction, binding pocket prediction, docking, similarity comparison, and binding affinity screening against hundreds to thousands of compounds in a shorter time, ensuring a comprehensive and automated analysis. Only compounds that exhibited a strong affinity, with Vina scores of −10 kcal/mol and below, were selected for further analysis and were further validated using Swissdock (http://www.swissdock.ch/) and PatchDock (http://bioinfo3d.cs.tau.ac.il/PatchDock/php.php) (accessed on 29 June 2023). Based on the initial data showing a strong binding affinity between binding pocket 3 and the established anthelminthic compounds, we proceeded to utilize the DrugReg tool for the screening of both FDA-approved drugs and naturally sourced compounds from the Traditional Chinese Medicine Library [39]. The Vina scoring function is a widely used empirical scoring function that estimates the binding affinity between a protein and ligand-based on their interaction energy and spatial complementarity. Selecting compounds with high binding affinity is crucial as it indicates a stronger potential for specific interactions with the target proteins. These compounds are more likely to exhibit favorable binding and potentially possess greater therapeutic efficacy against the target proteins. 

### 2.5. Analyses of the Binding Affinity of the SmGST Protein to the Compounds

In this study, we utilized computational tools to analyze and visualize the binding affinities of 27 compounds against the *Sm*GST proteins. The Vina scores of these compounds were computed and arranged in an Excel spreadsheet for further analysis. To create visually appealing data visualizations, we employed the Datawrapper tool (https://www.datawrapper.de/) (accessed on 9 August 2023), which is an online platform known for its user-friendly interface and interactive visualization capabilities. By inputting the Vina scores into Datawrapper, we generated a chart to represent the binding affinities of the 27 compounds against the target proteins. This visualization provided an overview of the compound–protein interactions and allowed for easy comparison and interpretation of the data. To further explore and illustrate this finding, we focused on visualizing the Vina scores of these 27 compounds specifically against the *Sm*GST protein. Using Datawrapper, we generated another chart that specifically highlighted the binding affinities of these compounds to *Sm*GST. 

### 2.6. Evaluation of Pharmacokinetic and Solubility Profiles for Scutiaquinone A and B

To assess the pharmacokinetic and solubility characteristics of Scutiaquinone A and B, we employed the SwissADME platform (http://www.swissadme.ch/) (accessed on 10 September 2023) [40]. SwissADME is an online resource specialized for applications in drug discovery and development, providing valuable data and prediction regarding the pharmacokinetic and physicochemical attributes of chemical compounds. This versatile tool scrutinizes essential parameters encompassing absorption, distribution, metabolism, and excretion, all of which significantly impact how compounds behave within the human body. We input the SMILES notation for Scutiaquinone A and B into SwissADME to compute their pharmacokinetics and solubility. The results, including lipophilicity, water solubility, drug-likeness, and other pharmacokinetic properties, were extracted and presented in a tabular form.

## 3. Results

**Figure 1:**
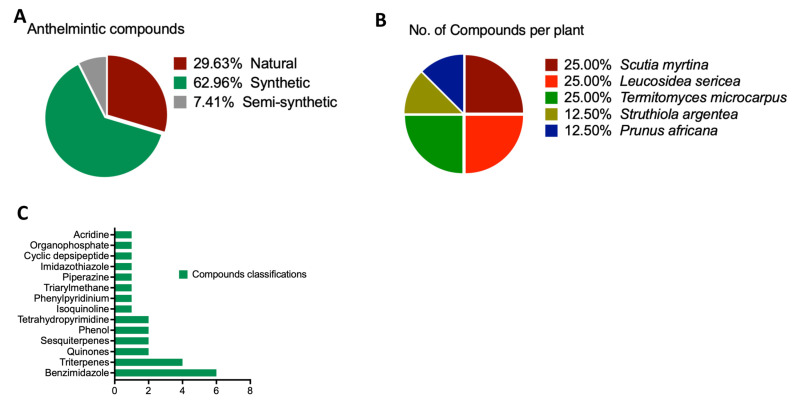
Scientific representation of compound characteristics. (**A**) The origin of anthelmintic compounds. (**B**) The botanical origins of the compounds. This aspect of the figure presents details about the particular plants from which the organic compounds are sourced. It emphasizes the wide range of botanical origins. (**C**) Categorization of the compounds.

A search across various databases was carried out to mine compounds with anthelmintic properties. Upon analyzing the data, we observed that anthelmintic compounds can be grouped into three main categories: natural compounds constituted 29.63% of the total, synthetic compounds made up 62.96%, and the remaining 7.41% comprised semi-synthetic compounds (Figure 1A). Among the natural compounds, a significant portion was derived from specific natural sources, with notable contributions stemming from *Scutia myrtina*, *Leucosidea sericea*, and *Termitomyces microcarpus* (Figure 1B). These sources likely harbor bioactive compounds known for their anthelmintic properties, rendering them appealing candidates for further exploration and potential drug developments. Additionally, the identified compounds were classified based on their chemical structures. This classification encompassed Quinones, Sesquiterpenes, and Triterpenes (Figure 1C). These categories represent distinct classes of organic compounds, each characterized by unique molecular structures and potentially novel mechanisms of action against helminth parasites. Conversely, synthetic compounds constituted the largest proportion of the identified anthelmintic compounds. The majority of these synthetic compounds belonged to the Sesquiterpenes category, followed by Phenol and Tetrahydropyrimidine, among others. Overall, this study’s findings shed light on the diversity of anthelmintic compounds originating from different sources, encompassing natural products derived from specific plants and chemically synthesized compounds. 

**Figure 2:**
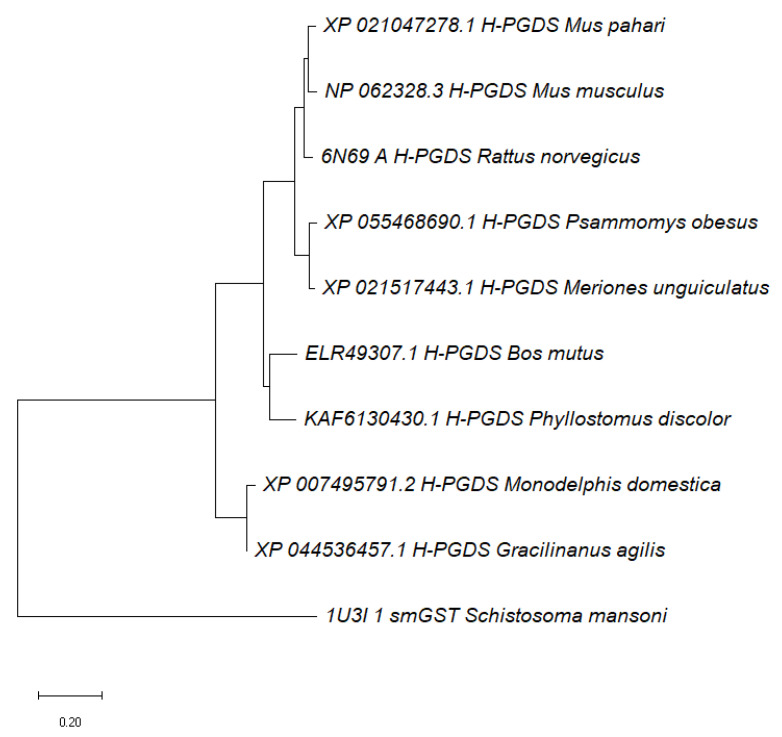
A phylogenetic tree depicting the distinct evolutionary divergence of the *Sm*GST from its mammalian homolog.

Upon conducting a protein BLAST analysis focused on mammalian proteins associated with *Sm*GST, we identified the protein hematopoietic prostaglandin D synthase isoform from *Bos mutus* (wild yak) as the closest match. This protein exhibited a sequence similarity of 36.13% to *Sm*GST. This indicates a distinction between *Sm*GST and its mammalian homologs, potentially implying that *Sm*GST could serve as a promising target for drug developments against *Schistosoma mansoni*. Furthermore, upon scrutinizing the constructed phylogenetic tree, it became evident that *Sm*GST exhibited a marked divergence from its analogous counterparts in humans and other mammalian species (Figure 2). Previous studies indicate that the search for novel potential drug targets has been focused on biochemical and metabolic pathways that show differences between pathogens and their hosts. 

**Figure 3:**
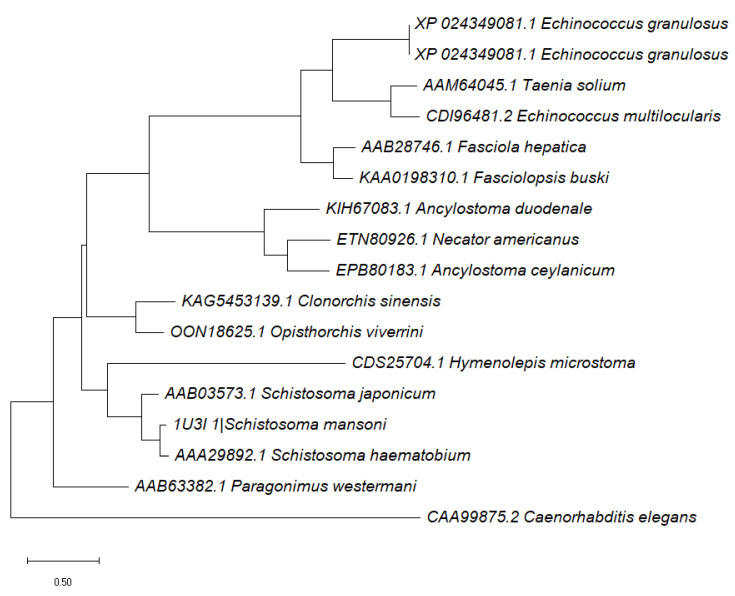
A phylogenetic tree depicting the close evolutionary relationship of the *Sm*GST protein to other parasitic worms in comparison to free-living worms.

The constructed phylogenetic tree illustrates a connection between *Sm*GST and various parasitic worms, including *Hymenolepis microstoma* and diverse *Schistosoma* species, emphasizing the shared ancestral lineage between *Sm*GST and other parasites that rely on animals as hosts. In contrast, the evolutionary origin of *C. elegans*, an organism that lives independently in the soil, deviates from these parasitic worms (Figure 3). The divergence in the phylogenetic placement of these two species reinforces the parasitic lifestyle of *Schistosoma mansoni* in contrast to the free-living nature of *C. elegans*. 

**Figure 4:**
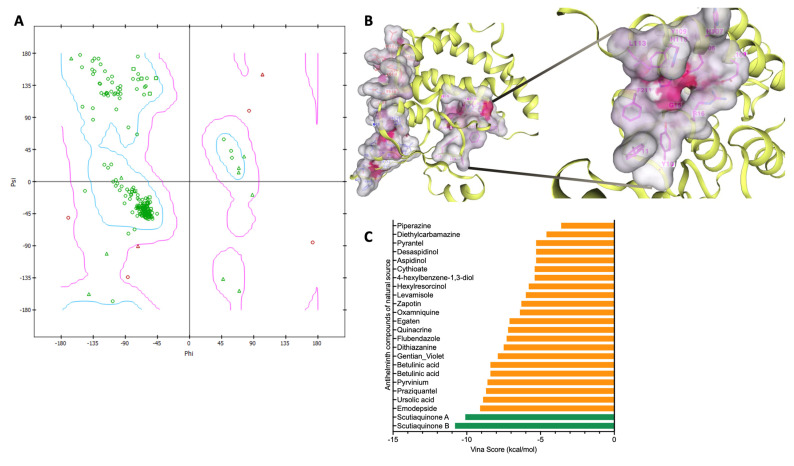
Binding sites and Vina score of the *Sm*GST protein and comparative anthelmintic compound affinities. (**A**). Ramachandran plot of the *Sm*GST protein showing the angles for each residue of the smGST. (**B**). Potential binding pockets of the *Sm*GST protein, with a focus on zooming out from pocket 3. (**C**). Bar chart illustrating the Vina score of the different anthelmintic compounds against the *Sm*GST protein. The bars highlighted in green were chosen for further analysis and discussion due to their Vina scores of less than −10 kcal/mol. The orange bars were not included as their Vina scores exceeded −10 kcal/mol.

**Figure 5:**
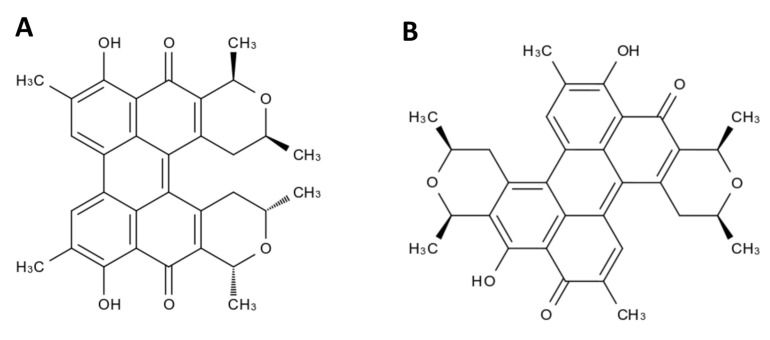
Scientific depiction of natural compounds (**A**) Scutiaquinone A and (**B**) Scutiaquinone B, derived from the South African Natural Compounds Database.

**Figure 6:**
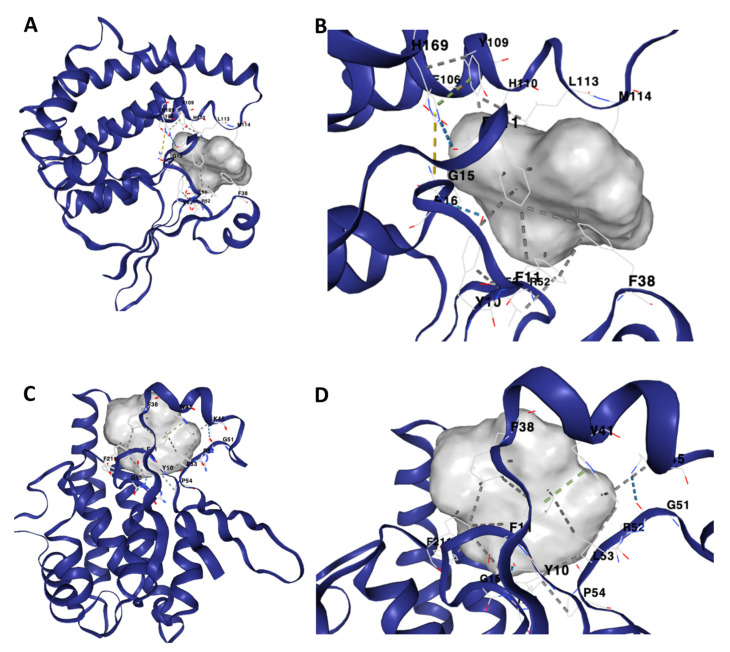
The three-dimensional binding structure of *Sm*GST protein shows its strong binding affinity to the two natural compounds. (**A**,**B**) Scutiaquinone A strongly binds to *Sm*GST and (**C**,**D**) Scutiaquinone B strongly binds to *Sm*GST. The compounds are shown in a gray-colored surface model, while the protein, *Sm*GST, is shown in a purple-colored cartoon model.

**Figure 7:**
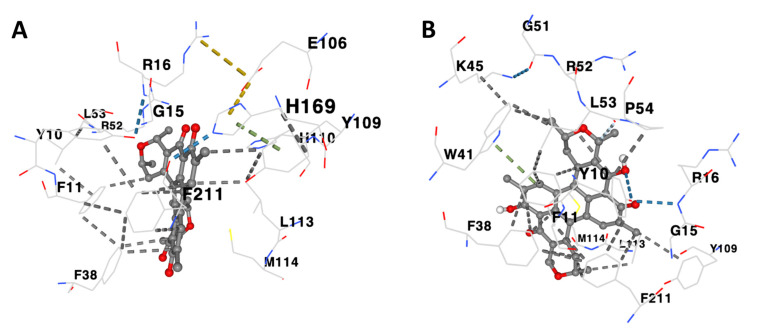
Residues of the *Sm*GST protein actively interacting with (**A**) Scutiaquinone A and (**B**) Scutiaquinone B. The interacting amino acid of *Sm*GST to Scutiaquinone A: TYR10 PHE11 GLY15 ARG16 PHE38 ARG52 LEU53 GLU106 TYR109 HIS110 LEU113 MET114 HIS169 PHE211. The interacting amino acid of *Sm*GST to Scutiaquinone B: TYR10 PHE11 GLY15 ARG16 PHE38 TRP41 LYS45 GLY51 ARG52 LEU53 PRO54 TYR109 LEU113 MET114 PHE211.

Table 1 illustrates the binding characteristics of distinct anthelmintic compounds against the GST proteins of *Schistosoma mansoni*. Prior to commencing the molecular docking experiment, we conducted an initial validation process for the three-dimensional structure of the *Sm*GST protein. To achieve this, we utilized the Ramachandran plot (Figure 4A). We further utilized DrugRep tools to conduct a comprehensive analysis of the binding pocket depicted in Figure 4B. Throughout the docking process, we methodically evaluated the binding affinity of compounds with the target proteins. Subsequently, for a more in-depth analysis emphasizing their binding strengths with *Sm*GST (Figure 4C), we exclusively selected compounds exhibiting robust affinity, characterized by Vina scores below −10 kcal/mol. Interestingly, our investigation revealed that none of the FDA-approved drugs or traditional Chinese medicine compounds displayed a Vina score lower than −10, suggesting a comparatively weaker or moderate binding affinity to the *Sm*GST protein. Consequently, we concentrated our scrutiny on anthelmintic compounds boasting Vina scores less than −10 kcal/mol with the *Sm*GST protein. Notably, the compounds exhibiting the most elevated binding affinities, as depicted in Figure 5, hold a higher potential for precise interactions with the target proteins, thereby potentially leading to enhanced therapeutic effects (Figure 6 and Table 1). Moreover, our findings underscore the robustness of the binding between the *Sm*GST protein and the natural compounds, Scutiaquinone A and B, as evidenced by the depiction of their respective interacting residues in Figure 7. Table 2 displays the pharmacokinetic and solubility profiles of Scutiaquinone A and B. Their drug-like properties were evaluated using Lipinski’s Rule of Five and Veber’s Rules. Both compounds meet these criteria, suggesting they have characteristics commonly found in pharmaceuticals and could be potential drug candidates. The pharmacokinetic data include factors like GI absorption, BBB permeability, P-glycoprotein substrate status, and interactions with various CYP enzymes. Notably, Scutiaquinone B exhibits enhanced GI absorption and is not a P-gp substrate, which could impact its transport in the body. However, both compounds exhibit poor water solubility, confirming their limited solubility and stability in methanol.

## 4. Discussion

In the current study, we explored various databases to identify anthelmintic compounds, which are drugs that inhibit the growth of parasitic worms. We searched for compounds that have been reported to have anthelmintic activity against *Schistosoma* or other helminths, or that share structural similarity with known anthelmintic drugs. Additionally, we conducted a molecular docking investigation involving these compounds and the *SmGST* protein. GSTs are enzymes that catalyze the conjugation of glutathione to xenobiotics, which are foreign substances that enter the body. This process helps to detoxify the xenobiotics and make them more soluble for excretion, making GSTs potential drug targets [41,42]. GSTs are widely distributed in living organisms, but they have different evolutionary origins and functions [43]. In *Schistosoma* parasites, GSTs play a crucial role in protecting them from oxidative stress and host immune responses [41,42].

Our search for antihelminth compounds and docking studies identified Scutiaquinone A and B as compounds that most likely possess antischistosomal activity. Scutiaquinone A and B are isolated from the roots of the *Scutia myrtina* plant, which is native to South Africa [44]. These compounds belong to a class of chemicals known as perylenequinones, a group of organic compounds that contain a perylene nucleus, which is a polycyclic aromatic system [45,46]. Interestingly, these compounds have previously demonstrated anthelmintic activities against *Haemonchus contortus* [44], a common and harmful parasite that infects the stomachs of ruminant animals like sheep and goats. It is a major cause of gastrointestinal parasitic infections in these animals, leading to significant economic losses in livestock industries worldwide [47]. Further, it has been shown that Scutiaquinone A and B possess complex chemical structures and diverse pharmacological properties, making them valuable starting points for drug discovery and development [44,46]. Based on our protein–ligand docking studies, Scutiaquinone A and Scutiaquinone B exhibited strong binding affinities to *Schistosoma mansoni* GST. Our results seem to corroborate the pharmacological effectiveness of Scutiaquinone A and B as lead compounds for drug discovery and development.

The pharmacokinetics and solubility profiles of Scutiaquinone A and B are presented, with an assessment of their drug-likeness based on Lipinski’s Rule of Five and Veber’s Rules, revealing that both compounds conform to these criteria [48]. This implies that Scutiaquinone A and B possess characteristics commonly observed in pharmaceuticals, suggesting their potential as viable drug candidates. The provided pharmacokinetic data encompass various parameters, including gastrointestinal (GI) absorption, blood–brain barrier (BBB) permeability, P-glycoprotein (P-gp) substrate status, and interactions with various cytochrome P450 enzymes (CYP) [49,50]. Notably, Scutiaquinone B demonstrates enhanced GI absorption, indicative of its propensity for absorption in the gastrointestinal tract.

Numerous studies have concentrated on finding therapeutic targets for enzymes with active sites that are distinct from those of their mammalian hosts and peculiar to helminths [51,52,53]. These studies suggest that parasite-specific metabolic pathways that demonstrate variations between helminths and their hosts can be the focus of the search for novel potential drug targets [51,53]. As a consequence, in our evolutionary analysis, we aimed to evaluate the distinctions between *Sm*GST and its homologs in other parasitic helminths as well as a representative free-living worm. The results of our work demonstrate that *Sm*GST had a common ancestor with other animal-parasitic worms, including *Hymenolepis microstoma* and several *Schistosoma* species. The free-living *C. elegans*, on the other hand, followed a different evolutionary path, showing a divergence from these parasitic worms. This may justify *Sm*GST as a more parasite-specific drug target than a host target.

Previously, several studies have employed computational methods to identify natural compounds and metabolites as potential inhibitors of various drug targets within the *Schistosoma mansoni* parasite [54,55,56,57,58,59]. In a recent study, a computational approach was employed to conduct a screening analysis involving several natural compounds sourced from medicinal plants indigenous to Saudi Arabia [54]. This screening utilized the docking technique, which serves to predict the optimal orientation and interactions between a ligand in this context, natural compounds, and a *Schistosoma mansoni* histone deacetylase 8 (*Sm*HDAC8) protein. Notably, the screening process unveiled nine previously undiscovered compounds demonstrating a robust binding affinity for *Sm*HDAC8. In another study, the researchers focused on *Sm*HDAC1, an enzyme belonging to the class I histone deacetylase (HDAC) category [55], which exhibits expression throughout all phases of the parasite’s life cycle and plays a vital role in the regulation of genes. In this screening process, they utilized a set of established class I HDAC inhibitors as molecular ligands and compared their affinities for binding to both *Sm*HDAC1 and *Hs*HDAC1 (human HDAC1), along with their respective concentrations required for inhibition. Their investigation revealed that a particular compound, namely, N,8-dihydroxy-8-(naphthalen-2-yl) octanamide (ZINC13474421), exhibited the highest level of selectivity and specificity for *Sm*HDAC1 in comparison to HsHDAC1. This observation suggests that ZINC13474421 holds promise as a potential candidate for further development as an anti-parasitic agent.

In our current study, we have employed a structure-based docking strategy to discern the anthelmintic potential of a selection of natural and synthetic compounds against various helminth parasites. This investigation entailed the computational screening of a compound library, primarily sourced from South Africa. Our findings have revealed that two distinct perylenequinone compounds, namely, Scutiaquinone A and B, exhibit robust binding affinities to a common binding site on SmGST proteins. The binding interactions between Scutiaquinone A and *Sm*GST primarily encompassed amino acid residues GLU106, HIS110, and HIS169, while Scutiaquinone B’s interactions predominantly involved residues TRP41, LYS45, GLY51, and PRO54. It is noteworthy that the majority of the interacting residues were shared by both compounds. The outcomes of our ongoing investigations strongly suggest the potential suitability of these two Perylenequinone compounds as druggable candidates for combating schistosomiasis.

## 5. Conclusions

In our study on natural Perylenequinone compounds as potential inhibitors of *Sm*GST, we address the important need for novel antischistosomal agents due to the limitations of the current treatment strategy relying on praziquantel. Our work focused on *Sm*GST, an essential parasite enzyme, and compared it to its human homolog, highlighting its distinctness. Our evolutionary analysis revealed that *Sm*GST shares a closer relationship with other parasitic worms than with free-living worms like *C. elegans*, emphasizing its potential as a drug target specific to parasites. Utilizing protein–ligand docking and binding pocket assessments, we identified Scutiaquinone A and Scutiaquinone B as robust binders to *Sm*GST. These naturally derived compounds, known for their effectiveness against similar parasites, demonstrate promising potential as antischistosomal agents. The unique characteristics of *Sm*GST make it a compelling target for drug discovery, and compounds like Scutiaquinone A and Scutiaquinone B offer hope for the development of alternative treatments for Schistosomiasis, addressing the urgent need for improved therapeutic options.

## Figures and Tables

**Table 1:** The binding affinity of the *Sm*GST protein and 27 anthelmintic compounds.

Database	Activities	S/N	Compound Name	Accession No	Sources	Class	Vina Score	Cavity Volume (Å3)	Center (x, y, z)	Docking Size (x, y, z)
SANCDB	**Anthelmintic Compound**	1	Scutiaquinone A	SANC00584	*Scutia myrtina*	Quinones	−10.1	297	16, 51, 34	22, 22, 22
		2	Scutiaquinone B	SANC00585	*Scutia myrtina*	Quinones	−10.8	297	16, 51, 34	23, 23, 23
		3	Aspidinol	SANC00741	*Leucosidea sericea*	Sesquiterpenes	−5.3	117	14, 56, 13	19, 19, 19
		4	Desaspidinol	SANC00742	*Leucosidea sericea*	Sesquiterpenes	−5.3	117	14, 56, 13	19, 19, 19
		5	Betulinic acid	SANC00743	*Termitomyces microcarpus*	Triterpenes	−8.4	117	14, 56, 13	22, 22, 22
		6	Zapotin	SANC01031	*Struthiola argentea*	Triterpenes	−6.3	117	14, 56, 13	21, 21, 21
		7	Betulinic acid	SANC00743	*Termitomyces microcarpus*	Triterpenes	−8.4	117	14, 56, 13	22, 22, 22
		8	Ursolic acid	SANC00744	*Prunus africana*	Triterpenes	−8.9	117	14, 56, 13	23, 23, 23
pubchem		9	4-hexylbenzene-1,3-diol	Compound CID: 3610	*Anacardium occidentale*, *Sargassum muticum*	Phenol	−5.4	117	14, 56, 13	20, 20, 20
drugbank		10	Albendazole	DB00518	Synthetic Source	Benzimidazole				
		11	Pyrantel	DB11156	Synthetic Source	Tetrahydropyrimidine	−5.3	117	14, 56, 13	20, 20, 20
		12	Piperazine	DB00592	Synthetic Source	Piperazine	−3.6	297	16, 51, 34	14, 14, 14
		13	Mebendazole	DB00643	Synthetic Source	Benzimidazole				
		14	Praziquantel	DB01058	Synthetic Source	Isoquinoline	−8.7	297	16, 51, 34	22, 22, 22
		15	Oxamniquine	DB01096	Semi-synthetic	Tetrahydroquinoline	−6.4	297	16, 51, 34	21, 21, 21
		16	Egaten	DB12245	Synthetic Source	Benzimidazole	−7.1	297	16, 51, 34	22, 22, 22
		17	Pyrvinium	DB06816	Synthetic Source	Phenylpyridinium	−8.6	297	16, 51, 34	24, 24, 24
		18	Gentian_Violet	DB00406	Synthetic Source	Triarylmethane	−7.9	297	16, 51, 34	23, 23, 23
		19	Diethylcarbamazine	DB00711	Synthetic Source	Piperazine	−4.6	117	14, 56, 13	18, 18, 18
		20	Levamisole	DB00848	Synthetic Source	Imidazothiazole	−6	117	14, 56, 13	19, 19, 19
		21	Hexylresorcinol	DB11254	Synthetic Source	Phenol	−5.8	297	16, 51, 34	20, 20, 20
		22	Emodepside	DB11403	Semi-synthetic	Cyclic depsipeptide	−9.1	117	14, 56, 13	29, 29, 29
		23	Flubendazole	DB08974	Synthetic Source	Benzimidazole	−7.3	297	16, 51, 34	23, 23, 23
		24	Cythioate	DB11392	Synthetic Source	Organophosphate	−5.4	297	16, 51, 34	20, 20, 20
		25	Quinacrine	DB01103	Synthetic Source	Acridine	−7.2	297	16, 51, 34	22, 22, 22
		26	Fenbendazole	DB11410	Synthetic Source	Benzimidazole				
		27	Dithiazanine	DB11516	Synthetic Source	Benzothiazole	−7.5	297	16, 51, 34	25, 25, 25

**Table 2:** The Pharmacokinetic and Solubility Profiles of Scutiaquinone A and B.

		Scutiaquinone A	Scutiaquinone B
**Lipophilicity**	Log Po/w (iLOGP)	4.38	4.39
	Log Po/w (XLOGP3)	5.72	6.17
	Log Po/w (WLOGP)	6.32	6.05
	Log Po/w (MLOGP)	2.62	2.62
	Log Po/w (SILICOS-IT)	6.68	6.71
	Consensus Log Po/w	5.15	5.19
**Water Solubility**	Log S (ESOL)	−6.88	−7.17
	Solubility	6.7 × 10^−5^ mg/mL; 1.31 × 10^−7^ mol/L	3.49 × 10^−5^ mg/mL; 6.83 × 10^−8^ mol/L
	Class	Poorly soluble	Poorly soluble
	Log S (Ali)	−7.44	−7.91
	Solubility	1.85 × 10^−5^ mg/mL; 3.62 × 10^−8^ mol/L	6.31 × 10^−6^ mg/mL; 1.24 × 10^−8^ mol/L
	Class	Poorly soluble	Poorly soluble
	Log S (SILICOS-IT)	−8.33	−8.11
	Solubility	2.39 × 10^−6^ mg/mL; 4.69 × 10^−9^ mol/L	3.96 × 10^−6^ mg/mL; 7.76 × 10^−9^ mol/L
	Class	Poorly soluble	Poorly soluble
**Druglikeness**	Lipinski	Yes	Yes
	Veber	Yes	Yes
	Bioavailability Score	0.55	0.55
**Pharmacokinetics**	GI absorption	Low	High
	BBB permeant	No	No
	P-gp substrate	No	No
	CYP1A2 inhibitor	No	No
	CYP2C19 inhibitor	Yes	No
	CYP2C9 inhibitor	Yes	Yes
	CYP2D6 inhibitor	No	No
	CYP3A4 inhibitor	Yes	No
	Log Kp (skin permeation)	−5.35 cm/s	−5.03 cm/s

## Data Availability

All data underlying the results are included as part of the published article.
